# Small caliber arterial endothelial cells calcium signals elicited by PAR2 are preserved from endothelial dysfunction

**DOI:** 10.1002/prp2.112

**Published:** 2015-02-02

**Authors:** John C Hennessey, Bruno D Stuyvers, John J McGuire

**Affiliations:** Cardiovascular Research Group, Division of BioMedical Sciences, Faculty of Medicine, Memorial UniversitySt. John's, Newfoundland, Canada

**Keywords:** Calcium imaging, Ca^2+^-release function, endothelial dysfunction, hypertension, protease-activated receptor 2, PAR-2, vascular endothelium

## Abstract

Endothelial cell (EC)-dependent vasodilation by proteinase-activated receptor 2 (PAR2) is preserved in small caliber arteries in disease states where vasodilation by muscarinic receptors is decreased. In this study, we identified and characterized the PAR2-mediated intracellular calcium (Ca^2+^)-release mechanisms in EC from small caliber arteries in healthy and diseased states. Mesenteric arterial EC were isolated from PAR2 wild-type (WT) and null mice, after saline (controls) or angiotensin II (AngII) infusion, for imaging intracellular calcium and characterizing the calcium-release system by immunofluorescence. EC Ca^2+^ signals comprised two forms of Ca^2+^-release events that had distinct spatial-temporal properties and occurred near either the plasmalemma (peripheral) or center of EC. In healthy EC, PAR2-dependent increases in the densities and firing rates of both forms of Ca^2+^-release were abolished by inositol 1,4,5- trisphosphate receptor (IP_3_R) inhibitor, but partially reduced by transient potential vanilloid channels inhibitor ruthenium red (RR). Acetylcholine (ACh)-induced less overall Ca^2+^-release than PAR2 activation, but enhanced selectively the incidence of central events. PAR2-dependent Ca^2+^-activity, inhibitors sensitivities, IP_3_R, small- and intermediate-conductance Ca^2+^-activated potassium channels expressions were unchanged in EC from AngII WT. However, the same cells exhibited decreases in ACh-induced Ca^2+^-release, RR sensitivity, and endothelial nitric oxide synthase expression, indicating AngII-induced dysfunction was differentiated by receptor, Ca^2+^-release, and downstream targets of EC activation. We conclude that PAR2 and muscarinic receptors selectively elicit two elementary Ca^2+^ signals in single EC. PAR2-selective IP_3_R-dependent peripheral Ca^2+^-release mechanisms are identical between healthy and diseased states. Further study of PAR2-selective Ca^2+^-release for eliciting pathological and/or normal EC functions is warranted.

## Introduction

Endothelial dysfunction is a factor of vascular pathophysiology and cardiovascular disease. Muscarinic receptors activation of endothelial cells (EC) by acetylcholine (ACh) is often used to assay vascular health status; decreased vasodilation by ACh is evidence of increased endothelial dysfunction (Triggle et al. 2012), although increased endothelium-dependent vasoconstriction is also reported (Feletou et al. 2011). In contrast, proteinase-activated receptor 2 (PAR2)-activating peptides maintain full efficacy for a normal endothelium-dependent vasodilation of small caliber arteries in animal models of hypertension, stroke, diabetes, and metabolic syndrome (McGuire et al. 2007; Smeda and McGuire 2007; Chia et al. 2011; Kagota et al. 2011; Howitt et al. 2014). PAR2 selectively activates Ca^2+^-activated potassium channels versus nitric oxide synthases in healthy small caliber arteries (McGuire et al. 2002, 2004a; McGuire 2004). PAR2 selective activation of Ca^2+^-activated potassium channels preserves vasodilator efficacy during endothelial dysfunction (Chia et al. 2011) and distinguishes the PAR2 mechanism from other EC receptors (e.g., muscarinic).

Ca^2+^-signals resulting from intracellular stores versus extracellular entry, in concert with subcellular organized Ca^2+^-signaling microdomains, differentiate the downstream signal transduction pathways of EC receptors (Ledoux et al. 2008b; Sandow et al. 2012; Sonkusare et al. 2012). Although PAR2 activation increases the global cytosolic Ca^2+^ in cultured EC (Klarenbach et al. 2003), the specific mechanisms and characteristics (e.g., spatial and temporal kinetics) of PAR2 Ca^2+^-release in single EC have not been investigated.

Our goal was to identify and characterize the mechanisms by which PAR2 activation produces intracellular Ca^2+^ signals in single EC of small caliber arteries. We aimed to determine the nature of the underlying PAR2 Ca^2+^-release function for EC in healthy and diseased states. Specifically, we tested the hypothesis that the PAR2 Ca^2+^-release function was selectively preserved from endothelial dysfunction in a mouse model of acquired hypertension. We compared EC from healthy mice to those with acquired hypertension, after chronic angiotensin II (AngII) infusion (McGuire et al. 2008; Chia et al. 2011). Our findings provide novel evidence showing two newly identified Ca^2+^ signals have the potential to link PAR2 activation in EC with restoring vasodilator function in blood vessels during endothelial dysfunction.

## Materials and Methods

### Animals

All animal care and experimental procedures were approved by the Institutional Animal Care Committee of Memorial University and conducted in accordance with the guidelines of the Canadian Council on Animal Care. Animals were provided free access to food, drinking water, and enrichment devices, and housed in rooms (12 h light/12 h dark periods) within a specific pathogen barrier facility.

52 animals in total were used in these studies. PAR2 knockout (KO) mice on C57BL/6J background (wild-type, WT) were from our colonies, which was established with original stock breeders from the Jackson Laboratories (C57BL/6J; B6.Cg-*F2rl*^tm1mslb^/J) as described (McGuire et al. 2008; Chia et al. 2011). The mice used for the experiments were third and fourth generation littermates of heterozygous PAR2 WT/KO breeders. Mice were randomly assigned to treatment groups, but the experimenter was not blinded to the treatment group.

### Mouse model of endothelial dysfunction after AngII infusion

Animals were administered saline (*n* = 26, vehicle controls, 0.25 *μ*L h^−1^) or AngII (*n* = 26; 1.5 mg kg^−1^ day^−1^ by continuous 14-day subcutaneous infusion via micro-osmotic pumps. Eleven to 13 weeks of age male littermates (WT, *n* = 26; KO, *n* = 26) weighing 22–33 g, were anesthetized with 2% isoflurane and oxygen (2 L min^−1^) and kept warm at 37°C on a heating platform during the surgeries (10–15 min) for implanting pump (model 1002; Alzet, Cupertino, CA) as described (McGuire et al. 2008; Chia et al. 2011).

### Live cell Ca^2+^ imaging by 2D confocal microscopy

On the mornings of experiment days, EC were isolated by enzyme dissociation from small mesenteric arteries (first-, second-, and third-order branches) of mice killed by cervical dislocation, after overdose inhalation of 100% isoflurane.

#### Cell preparation

Exposure to trypsin-like serine protease activity, which could desensitize PAR2, was avoided by selection of appropriate grades of reagents. Briefly, mesenteric arterial cascades were removed from heparinized (10 units) mice and arterial branches excluding the superior-mesenteric artery were dissected free of adherent tissues in ice-chilled EC isolation buffer (ECIB; pH 7.4; mmol/L: NaCl, 55; sodium glutamate, 80; KCl, 6; MgCl_2_, 2; CaCl_2_, 1; 4-(2-hydroxyethyl)-1-piperazineethanesulfonic acid hemisodium salt [HEPES], 10; glucose, 10) (Ledoux et al. 2008a). Arterial branches were finely chopped, and incubated 5 min at 4°C in vascular smooth muscle cell (VSMC) isolation buffer containing no Ca^2+^ (pH 7.4; mmol/L: NaCl, 55; sodium glutamate, 80; KCl, 5; MgCl_2_, 2; ethylene glycol tetraacetic acid [EGTA], 0.1; HEPES, 10; glucose, 10) (Luykenaar and Welsh 2007). Samples incubated with dithiothreitol (0.5 mg mL^−1^) and papain (0.5 mg mL^−1^; Worthington Biochemical, Lakewood, NJ) for 30 min at 37°C, and low amounts of CaCl_2_ (0.2 mmol/L), collagenase IV (1.0 mg mL^−1^; Worthington Biochemical) and neutral protease (1.0 mg mL^−1^; Worthington Biochemical) for 25 min at 37°C. Protease activities were stopped by rapidly cooling samples on ice, and diluting the reaction mixture with ECIB containing 2 mmol/L CaCl_2_ (ECIB-Ca^2+^). Suspensions of EC and VSMC were generated by titurating samples, washed twice by centrifuging, and resuspending cell pellets in ECIB-Ca^2+^. Suspensions were kept on ice until used for Ca^2+^ imaging or immunofluorescence experiments. This method yielded 150–200 EC per mouse. EC were distinguished from VSMC by their distinct appearances (cell shapes and sizes) when viewed in different *z*-planes using bright field and confocal fluorescence microscopy and by the lack of platelet endothelial cell adhesion molecule-1 (PECAM-1) detection in immunofluorescence studies.

#### Protocols

The first series of Ca^2+^ imaging experiments in EC were performed with no agonist (baseline), and a concentration range of agonists (2-furoyl-LIGRO-amide purchased from Peptide Synthesis Facility [University of Calgary, AB] [McGuire et al. 2004b], 2fly, 0.1 nmol/L to 3 *μ*mol/L; ACh, 1 nmol/L to 30 *μ*mol/L), in the absence of inhibitors of Ca^2+^-release channels. To control for variation between animals (within genotype and pump treatment) for agonist-mediated responses, three of the five to six runs collected internal reference data in every animal: baseline, 30 nmol/L 2fly, and 300 nmol/L ACh; the other runs collected data for one of a range of concentrations of 2fly, and ACh. In the second series, experiments were performed in the absence, and presence of inhibitors without agonists (baseline) and with single mid-range effective concentrations of agonists (2fly, 30 nmol/L; ACh, 300 nmol/L).

In the first series of experiments, test compounds in ECIB-Ca^2+^ were superfused for 1 min, buffer flow stopped and Ca^2+^-imaging data acquired. In the second series, inhibitors in ECIB-Ca^2+^ were superfused for 1 min, buffer flow stopped for 10 min, test compounds added directly to cells, and Ca^2+^-imaging data acquired.

#### Ca^2+^ imaging

Cells incubated with Fluo4-AM (10 *μ*g mL^−1^) at room temperature for 10 min were transferred to 35 mm uncoated Fluorodish (World Precision Instruments, Sarasota, FL), allowed to settle from suspension (adhere to the bottom) and washed with ECIB-Ca^2+^ (gassed with 95% O_2_/5% CO_2_) at 37°C for 10 min. These washed preparations were placed in the experimental chamber of an inverted motorized microscope (Olympus IX81; Olympus Canada, Richmond Hill, ON) equipped with a confocal Nipkow spinning disk unit (CSU-X1; Yokogawa, Tokyo, Japan) and superfused with O_2_-bubbled ECIB-Ca^2+^ at 37°C.

Intracellular Ca^2+^ dynamics were recorded by 2-dimension (2D) CM (Haq et al. 2013). Briefly, Ca^2+^ events were measured as elevations of Ca^2+^-Fluo4 fluorescence along the mid planar region of EC. Fluo-4 loaded cells illuminated at 488 nm (FRAP-3D MAG Biosystems; Photometrics, Tuscon, AZ) emitted fluorescence filtered at 512 nm and collected by a low light sensitive CCD camera (Rolera-MGI Plus; Q Imaging Systems, Surrey, BC). 2D full field snapshots of instantaneous fluorescence were taken at video rate (30 frames sec^−1^) and converted into 512 × 512 pixels resolution images. Stacks of 300 frames (∼10 sec) were converted into ratio (*F*/*F*_0_) images by dividing pixel-to-pixel each (*F*) image by the reference (*F*_0_) image selected before the Ca^2+^ variation of interest. Offline image processing was performed using the NIH (Bethesda, MD) software *ImageJ*.

#### Image analyses

Briefly, Ca^2+^ dynamics were analyzed from *F*/*F*_0_-image stacks. The stacks were inspected visually, Ca^2+^-release sites identified, and counted; the number of Ca^2+^ events was estimated per site over 10 sec periods. Spatial properties of Ca^2+^ events were determined from *F*/*F*_0_-images by measuring the pixel (*voxel*) profiles along virtual line-scans in the *x*/*y* directions; time-course of Ca^2+^ events estimated by measuring variations of the pixel (*voxel*) profiles through the stacks.

### Immunocytochemistry

We modified a method (Stuyvers et al. 2005) to prepare EC. Briefly, cells were fixed with formaldehyde (1.26% w/v) in phosphate buffered solution (PBS; pH 7.4 at 4°C; mmol/L: NaCl, 137; KCl, 2.7; Na_2_HPO_4_, 10; KH_2_PO_4_, 2.0), and permeabilized with 0.1% saponin in PBS containing 3% (w/v) bovine serum albumin, for blocking non-specific protein binding by antibodies. Fixed permeabilized cells were centrifuged (120*g*) for 5 min, resuspended in PBS, and incubated overnight with PECAM-1 polyclonal goat antibody (1:500 dilution) and a selected target antibody at 4°C. Cells were washed with PBS and incubated for 90 min at 4°C with Texas Red® dye-conjugated AffinPure Bovine anti-goat IgG (1:500 dilution, 805-075-180) and fluorescein isothiocyanate (FITC)-conjugated AffinPure Goat anti-rabbit IgG (1:1000 dilution; 111-095-003,); both purchased from Jackson ImmunoResearch Laboratories (West Grove, PA).

Rabbit polyclonal antibodies were used to detect PAR2, endothelial or nitric oxide synthase 3 (eNOS), small-conductance Ca^2+^-activated potassium channel KCNN3 (K_Ca_2.3) and intermediate-conductance Ca^2+^-activated potassium channel KCNN4 (K_Ca_3.1); all are known to label these proteins in the mouse. Rabbit antibody for PAR2, B5 (Kong et al. 1997), was provided by Dr. Morley Hollenberg (University of Calgary, AB) and used at 1:1000 dilution. Antibodies for eNOS (1:100 dilution; ab66127), K_Ca_2.3 (1:2000 dilution; ab83737), and K_Ca_3.1 (1:2000 dilution; ab83740) were purchased from Abcam (Cambridge, MA). Mouse antibody for human inositol 1,4,5-trisphosphate receptor (IP_3_R) type 1 (IP_3_R1; 1:1000; cat# 407140) was purchased from EMD Millipore (Etobicoke, ON); the targeted amino acids sequence (22–230) is nearly identical with mouse IP_3_R1 and very highly conserved with mouse IP_3_R2 and IP_3_R3.

Standard (laser scanning) fluorescence confocal microscopy (CM) (Olympus Fluoview 1000) detected antibodies. 2D line scan images were sampled at 1024 × 1024 pixels resolution. Co-localization experiments used dual excitation (488 nm [FITC], 512 nm [Texas Red]; sequential mode scan). Vertical optical slicing (*z*-steps: 0.25 *μ*m) assessed cell volume distributed fluorescent antibodies.

Virtual line scans across the mid-plane of EC assessed protein distribution. Line scan data were expressed (in grey scale) as percentage of Maximal Fluorescence Intensity. In space, the fluorescence distribution was characterized across the full cell width and normalized to % Max Cell Width. Signal intensity for immunoreactive protein expression data were expressed as area-under-the-curve for statistical comparisons between groups.

## Materials

Unless otherwise indicated general reagents and chemicals were purchased from Sigma-Aldrich (Oakville, ON).

### Statistical analyses

Data are presented as mean ± SEM. Student unpaired *t*-tests, and ANOVA with Bonferroni post hoc test were used to compare variables as appropriate. *P *<* *0.05 was considered significant.

## Results

### Identification and characterization of PAR2 Ca^2+^-release events in EC of small caliber arteries in the healthy state

**Figure 1:**
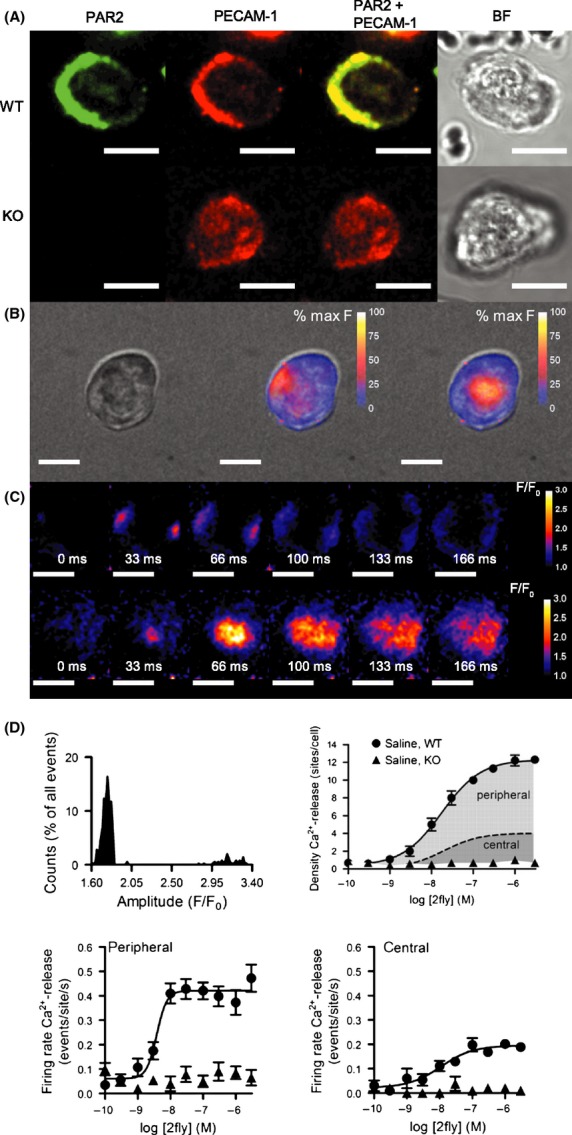
Identification and characterization of PAR2 Ca^2+^-release events in EC of small caliber arteries in the healthy state. (A) Immunofluorescence detection of PAR2 alone (green), platelet endothelial cell-adhesion molecule (PECAM-1) alone (red), PAR2 merged with PECAM-1 and bright field (BF) images in EC from PAR2 WT and KO mice, after 14 days infusion with saline. Fixed permeabilized small caliber mesenteric arterial EC incubated with B5- and PECAM-1 primary, and FITC- and Texas Red-conjugated secondary antibodies. Yellow indicates overlap of PAR2 and PECAM-1. (B) Fluo4-Ca^2+^ fluorescence 2D spinning disk CM data superimposed on BF image of EC during exposure to PAR2 agonist 2fly (3 *μ*mol/L). Two-dimensional raw instantaneous fluorescence (*F*) data (30 frames sec^−1^) were converted and calibrated to 8-bit gray scales (255 = 100%). Peripheral (middle column) and central (right column) events are shown at the peak amplitude. (C) Ratio (*F/F*_0_) images of the time- and space-courses for peripheral (upper row) and central (lower row) events in EC exposed to 2fly. EC is the same cell shown in B. Ratio images were calculated by dividing a reference frame (*F*_0_) into the subsequent frames (*F*). Each frame in the time-courses follows after *F*_0_. In the upper row, two events with separated peripheral origins are shown occurring simultaneously. (D) Frequency distribution of the measured peak amplitudes of all Ca^2+^ events (*N* = 303) elicited by 2fly (3 *μ*mol/L) in *n *=* *10 cells isolated from saline WT EC. Amplitude bin size was 0.02 *F/F*_0_. Concentration-response data for PAR2-activating peptide 2fly in EC (*n *=* *10 cells/point; 100 cells for each curve) from WT and KO. Density of total Ca^2+^-release events in WT EC exposed to 2fly is the sum of the area shaded dark gray under dashed line indicating the number of central events and the area shaded light gray above dashed line indicating number of peripheral events. Firing rates of the peripheral and central events in WT and KO EC exposed to 2fly. Data were acquired as described in B–C in separate EC. For all images white bar = 10 *μ*m.

To validate the protocol for isolated cell preparations, we measured PAR2 and EC-specific marker PECAM-1 immunofluorescence in fixed permeabilized EC. Saline WT (control) EC expressed PAR2 and PECAM-1. PAR2 immunofluorescence detection by B5-antibody co-localized with PECAM-1 near the plasmalemma, and heterogeneously spread within the cytoplasm in saline WT EC (Fig.1A). B5-antibody did not bind to saline KO EC, which positively stained for PECAM-1 (Fig.1A). Based on the comparison of group data, PECAM-1 immunofluorescence was not different between WT and KO EC.

To identify PAR2 Ca^2+^-release mechanisms in EC of small caliber arteries in a healthy state, intracellular Ca^2+^ dynamics was assessed in saline WT EC. In saline WT EC (Fig.1B left) exposed to PAR2-activating peptide 2fly, two types of Ca^2+^-transients were observed: (1) events occurring near the plasmalemma (Fig.1B middle) and (2) events occurring near the center (Fig.1B right). Ratio image (*F*/*F*_0_) analyses of events at peripheral (Fig.1C top) and central (Fig.1C bottom) sites indicated distinct unique spatial and temporal kinetic properties, summarized in Table1. Frequency distribution of the peak amplitudes for all events in saline WT EC, exposed to 2fly, confirmed the existence of two separate groups in the population of events, each group matching with individual sites localization in EC; see Figure1D, mean peak amplitude (*F*/*F*_0_) peripheral versus central: 1.77 ± 0.01 versus 3.14 ± 0.02; majority of PAR2 Ca^2+^-release (88% of total events) occurred at peripheral sites.

**Table 1:** Spatial and temporal kinetic properties of peripheral and central Ca^2+^-release events in EC from small caliber mesenteric arteries of PAR2-WT and PAR2-KO mice, after saline or AngII infusions in vivo, measured in absence (baseline) and presence of PAR2-activating peptide (2fly, 3 *μ*mol/L), or ACh (30 *μ*mol/L)

Genotype/Treatment	Drug	Events (*N*)	Amplitude (*F*/*F*_0_)	FWHM (% Max Cell Width)	*t*_rise_ (msec)	*t*_1/2_ (msec)	Frequency (Hz)
Peripheral events							
PAR2-WT							
Saline	Baseline	10	1.80 ± 0.03	20 ± 1	80 ± 2	203 ± 3	N/A
	2fly	303	1.77 ± 0.01	20 ± 1	80 ± 1	205 ± 1	N/A
	ACh	137	1.78 ± 0.02	20 ± 1	80 ± 1	206 ± 1	N/A
AngII	Baseline	5	1.78 ± 0.04	22 ± 2	81 ± 3	205 ± 3	N/A
	2fly	289	1.77 ± 0.01	20 ± 1	80 ± 1	204 ± 1	N/A
	ACh	92*	1.80 ± 0.02	20 ± 1	80 ± 1	204 ± 1	N/A
PAR2-KO							
Saline	Baseline	9	1.81 ± 0.03	20 ± 2	78 ± 2	203 ± 2	N/A
	2fly	9	1.75 ± 0.03	19 ± 1	78 ± 3	203 ± 3	N/A
	ACh	126	1.76 ± 0.01	20 ± 1	80 ± 1	205 ± 3	N/A
AngII	Baseline	6	1.79 ± 0.05	19 ± 2	79 ± 3	203 ± 2	N/A
	2fly	7	1.76 ± 0.04	20 ± 2	81 ± 2	206 ± 2	N/A
	ACh	93*	1.78 ± 0.03	20 ± 1	81 ± 1	206 ± 1	N/A
Central events							
PAR2-WT							
Saline	Baseline	4	3.11 ± 0.06†	33 ± 1†	101 ± 4†	113 ± 4†	0.02 ± 0.02
	2fly	45	3.14 ± 0.02†	34 ± 1†	98 ± 1†	115 ± 1†	0.64 ± 0.12‡§
	ACh	63	3.12 ± 0.02†	33 ± 1†	99 ± 1†	115 ± 1†	0.82 ± 0.18§
AngII	Baseline	3	3.19 ± 0.05†	31 ± 1†	102 ± 3†	112 ± 1†	0.02 ± 0.02
	2fly	43	3.14 ± 0.02†	33 ± 1†	101 ± 1†	115 ± 1†	0.52 ± 0.11‡§
	ACh	30*	3.14 ± 0.02†	33 ± 1†	99 ± 2†	115 ± 1†	0.32 ± 0.06*§
PAR2-KO							
Saline	Baseline	3	3.17 ± 0.05†	31 ± 2†	93 ± 2†	116 ± 5†	0.03 ± 0.02
	2fly	2[Table-fn tf1-1]	3.16 ± 0.07†	32 ± 1†	99 ± 4†	112 ± 2†	0.02 ± 0.01
	ACh	65	3.14 ± 0.02†	33 ± 1†	100 ± 1†	116 ± 1†	0.77 ± 0.06§
AngII	Baseline	3	3.21 ± 0.05†	33 ± 2†	102 ± 2†	112 ± 4†	0.02 ± 0.02
	2fly	4	3.11 ± 0.12†	36 ± 1†	99 ± 5†	117 ± 2†	0.04 ± 0.02
	ACh	33*	3.13 ± 0.02†	33 ± 1†	100 ± 2†	113 ± 2†	0.34 ± 0.04*§

*N*, total events observed in 10 cells; N/A, not applicable. Temporal and spatial kinetic parameters are mean ± SE for *n* = 10 cells (*N* events), except for^a^, where *n* > 10 cells to observe 3 central events in KO.

*P <* *0.001, AngII vs. saline two-way ANOVA (genotype × treatment) followed by Bonferroni post hoc test; ^†^*P *<* *0.001, central vs peripheral; ^‡^*P *<* *0.001, WT vs. KO; and §*P *<* *0.001, agonist vs. baseline: two-way ANOVA (genotype × treatment) followed by the Bonferroni post hoc test.

From Table1, peak amplitudes and four additional characteristics differentiated the types of events. Full width at half maximum of the transient (FWHM) estimated the spread of Ca^2+^-release and was 70% larger (*P *<* *0.05) for central versus peripheral events. The rising phase of the transient (time-to-rise [*t*_rise_] from baseline to maximum amplitude) was 18% faster (*P *<* *0.05) for peripheral than central events. The transient decline (time-to-fall [*t*_1/2_] from maximum amplitude to half maximum amplitude) was 1.8-times slower (*P *<* *0.05) for peripheral than central events. Central events occurred at constant frequency (0.64 ± 0.12 Hz) in the presence of 2fly, and originated from the exact same loci in the cells.

In saline WT EC, 2fly elicited a concentration-dependent increase in the density (number of sites/cell; Fig.1D), and in the firing rates (events/site per sec; Fig.1D) for peripheral, and central events. In saline KO EC, 2fly increased neither density nor firing rates of Ca^2+^ events which remained same as saline WT EC at basal (untreated) conditions. In saline WT and KO EC, central and peripheral events characteristics (Table1) at baseline were not different than with 2fly, except for firing rate of central Ca^2+^-release sites which was larger in WT.

### Characterization of PAR2 Ca^2+^-release in EC of small caliber arteries in healthy and diseased states

**Figure 2:**
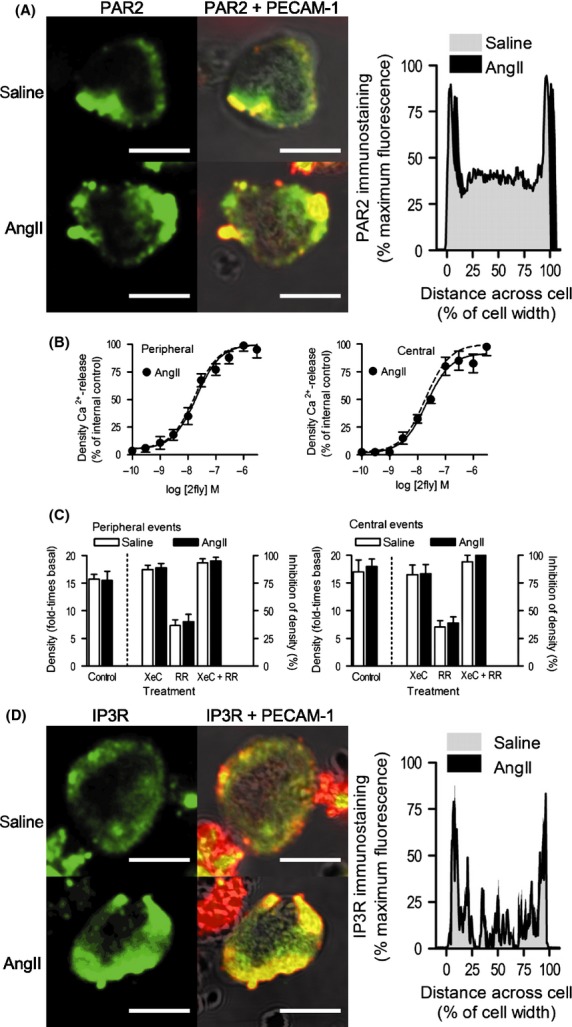
Characterization of PAR2 Ca^2+^-release mechanism in EC of small caliber arteries in healthy and diseased states. (A) Immunofluorescence detection of PAR2 alone (green), and PAR2 merged with PECAM-1 superimposed on BF images (red, PECAM-1; yellow, PAR2 + PECAM-1 overlap) in EC from *par2* wild-type (WT) mice, after 14 days infusion with vehicle (saline) or AngII. Fixed permeabilized small caliber mesenteric arterial EC incubated with B5- and PECAM-1 primary, and FITC- and Texas Red-conjugated secondary antibodies. Saline and AngII WT EC (*n *=* *6 cells per group) PAR2 expression were quantified by averaging virtual line scans of pixel fluorescence across the center planes of a *z*-stack image series (0.25 *μ*m steps). Lines bounding gray (saline) and black (AngII)-shaded areas represent the mean standardized fluorescence across the normalized cell widths; AngII group has been right shifted on *y*-axis to show the data. (B) Concentration-response data for PAR2-activating peptide 2fly in AngII WT EC. Densities of peripheral and central Ca^2+^-release events in WT EC exposed to 2fly were normalized to mean maximum responses reported in the WT saline group; solid and dashed lines indicate best-fit curves for normalized AngII (*n *=* *10 cells/point; 100 cells per curve) and saline groups (Fig.1D), respectively. Fluo4-Ca^2+^ fluorescence data were acquired and analyzed as outlined in Figure1. (C) Effects of IP_3_R inhibitor, XeC, and TRPV inhibitor, RR, on PAR2 Ca^2+^-release. In WT saline and AngII EC exposed to 2fly (30 nmol/L), Ca^2+^-release data were recorded in the absence (control), and presence of XeC (2 *μ*mol/L), RR (75 *μ*mol/L), and XeC + RR (*n *=* *10 cells/treatment). Positive increases in densities (controls) are reported on left *y*-axes for peripheral and central events (fold-times basal (no agonist) conditions). Inhibitions of Ca^2+^-release densities by pretreatments are reported on right *y*-axes. (D) Immunofluorescence detection of IP_3_R alone (green), and IP_3_R merged with PECAM-1 superimposed on BF images (red, PECAM-1; yellow, IP_3_R + PECAM-1 overlap) in WT saline, and AngII EC. Fixed permeabilized EC from small caliber mesenteric arteries incubated with IP_3_R- and PECAM-1-primary, and FITC- and Texas Red-conjugated secondary antibodies. Saline (*n *=* *6) and AngII (*n *=* *6) WT EC IP_3_R expressions were quantified as described for PAR2. For all images white bar = 10 *μ*m.

To determine PAR2 expression in the isolated EC of small caliber arteries in the healthy and diseased states, we quantified B5-antibody immunofluorescence in 2D confocal images across the mid-plane of EC. PAR2 co-localized with PECAM-1 near the plasmalemma, and spread heterogeneously in the cytoplasm of WT saline and AngII EC (Fig.2A). PAR2 in saline WT was not distributed differently than in AngII WT EC (Fig.2A).

To identify the PAR2 Ca^2+^-release mechanism in EC from small caliber arteries in a diseased state, intracellular Ca^2+^ dynamics was assessed in AngII WT EC by 2D CM. The same two types of events observed in saline WT were observed in AngII WT EC. Peripheral and central events characteristics (Table1) in AngII WT EC exposed to 2fly were not different than in saline WT EC. In AngII WT EC, 2fly caused concentration-dependent increases (Fig.2B) in the density of peripheral and central events. The diseased state of small caliber arteries in AngII WT had no effect on PAR2 Ca^2+^-release function (raw data were normalized by saline WT maximum responses (Fig.1D) to highlight this point). Similarly, increases in firing rates of peripheral and central events by 2fly in AngII WT (data not shown) were not different than in saline WT EC.

In AngII WT and KO EC, central and peripheral events characteristics (Table1) at baseline were not different than with 2fly present.

To identify the molecular nature of the Ca^2+^-release units underlying PAR2 mechanisms in EC of arteries in healthy and diseased states, Ca^2+^ dynamics in EC pretreated with vehicle (control) were compared to those pretreated with xestospongin C (XeC) (IP_3_R inhibition), ruthenium red (RR) (transient receptor potential vanilloid channel [TRPV] inhibition), and XeC + RR (IP_3_R and TRPV inhibition). In saline WT EC, 2fly (at EC_50_) increased the peripheral events (Fig.2C left) density by 15-times basal (left *y*-axis). This increase was nearly abolished by XeC, partly reduced by RR, and blocked by XeC + RR (right *y*-axis). Similarly in saline WT EC, 2fly increased the central events (Fig.2C right) density by 16-times basal (left *y*-axis) and this increase was nearly abolished by XeC, partly reduced by RR, and blocked by XeC + RR (right *y*-axis). This inhibition by XeC, RR, and XeC + RR of peripheral and central events densities was identical in WT AngII and saline WT EC.

To characterize the expression of IP_3_R in the isolated EC of small caliber arteries in the healthy and diseased states, we measured the IP_3_R-antibody fluorescence intensity across the mid-plane of EC. IP_3_R colocalized with PECAM-1 at the periphery and spread heterogeneously throughout the cytoplasm in saline WT and AngII WT EC (Fig.2D). IP_3_R in saline WT was not distributed differently than in AngII WT EC (Fig.2D).

### Characterization of muscarinic receptors-mediated Ca^2+^-release mechanism in EC of small caliber arteries in healthy and diseased states

**Figure 3:**
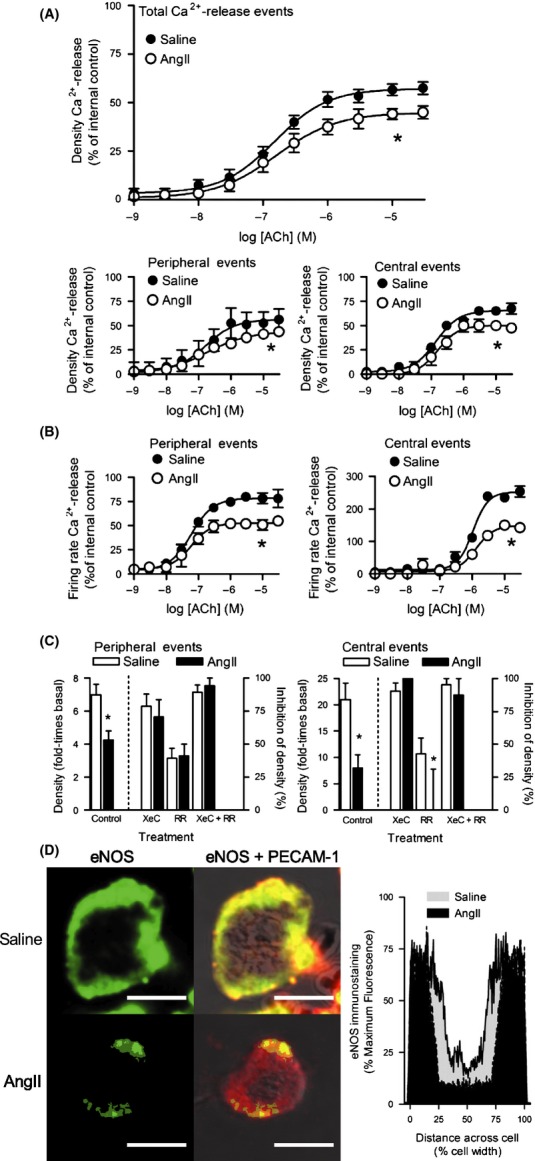
Characterization of muscarinic receptors mediated Ca^2+^-release mechanism in EC of small caliber arteries in healthy and diseased states. (A–B) Concentration-response data for ACh in EC from PAR2 WT mice, after 14 days infusion with vehicle (saline) or AngII. Densities and firing rates for total, peripheral, and central Ca^2+^-release events in WT EC were normalized to mean maximum responses by 2fly in WT saline group (Fig.1D); *n *=* *10 cells/point, 100 cells per curve for each group. Fluo4-Ca^2+^ fluorescence data were acquired and analyzed as outlined in Figure1. *P <* *0.01, E_max_, saline vs. AngII. (C) Effects of IP_3_R inhibitor, XeC, and TRPV inhibitor, RR, on muscarinic receptors Ca^2+^-release mechanism. In saline and AngII WT EC exposed to ACh (300 nmol/L), Ca^2+^-release data were recorded in the absence (control), and presence of XeC (2 *μ*mol/L), RR (75 *μ*mol/L), and XeC + RR (*n *=* *10 cells/treatment). Positive increases in densities (controls) are reported on left *y*-axes for peripheral and central events (fold-times basal (no agonist) conditions). Inhibitions of Ca^2+^-release densities by pretreatments are reported on right *y*-axes. *P <* *0.01, control, and RR: saline vs. AngII. (D) Immunofluorescence detection of eNOS alone (green), and eNOS merged with PECAM-1 superimposed on BF images (red, PECAM-1; yellow, eNOS + PECAM-1 overlap) in WT saline and AngII EC. Fixed permeabilized EC from small caliber mesenteric arteries incubated with eNOS-, PECAM-1- primary antibodies, and FITC- and Texas Red-conjugated secondary antibodies. WT saline (*n *=* *6) and AngII EC (*n *=* *6) eNOS expressions were quantified as described for PAR2 in Figure1. Lines bounding gray (saline) and black (AngII) shaded areas represent the mean standardized fluorescence across the normalized cell widths. *P <* *0.05, Area-under-the-curve, saline vs. AngII. White bar=10 *μ*m.

To further identify and characterize Ca^2+^-release mechanisms in EC of small caliber arteries in a healthy state, Ca^2+^ dynamics elicited through activation of muscarinic receptors was assessed in saline WT EC. ACh caused a concentration-dependent increase in the density of total Ca^2+^-release events (Fig.3A). Spatial-temporal kinetic properties of these events were not different than those events in 2fly-exposed EC observed at baseline in WT and KO (Table1). At 30 *μ*mol/L ACh, a larger proportion of events occurred centrally than was seen with 3 *μ*mol/L 2fly (31% vs. 12%). However, the majority of Ca^2+^-release still occurred along the cell periphery. ACh concentration-dependent increases in the densities of total, peripheral, and central Ca^2+^-release events reached plateaus of 50–60% of the maximum effects by PAR2 (raw data for Fig.3A–B was normalized relative to the maximum effect by 2fly (Fig.1D) in order to highlight the differences). ACh concentration-dependent increases in firing rates of peripheral and central events reached plateaus that were 75%, and 200%, respectively, of the maximum by PAR2 (Fig.3B).

To identify and characterize Ca^2+^-release mechanisms in EC of small caliber arteries in a diseased state, Ca^2+^ dynamics elicited by ACh was assessed in AngII WT EC. ACh concentration-dependent increases in the densities of total, peripheral, and central Ca^2+^-release in AngII WT EC were 25% lower relative to controls (Fig.3A). Similarly, the AngII WT EC firing rates of peripheral and central events were decreased by 33% and 50%, respectively, relative to controls (Fig.3B).

To identify the molecular nature of the Ca^2+^-release units underlying the muscarinic mechanisms in EC of arteries in healthy and diseased states, Ca^2+^ dynamics in EC pretreated with vehicle (control) were compared in EC exposed to XeC, RR, and XeC + RR. In saline WT EC, ACh at EC_50_ increased the peripheral events (Fig.3C left) density by seven times (left *y*-axis) and was inhibited by 80% with XeC, 30% with RR, and 90% with XeC + RR (right *y*-axis). Similarly in saline WT EC, ACh at EC_50_ increased the central events (Fig.3C right) density by 20-times (left *y*-axis) and was nearly abolished by XeC, partly reduced by RR, and blocked by XeC + RR (right *y*-axis).

ACh induced less Ca^2+^-release in WT AngII than in saline EC. In AngII WT EC, ACh increased the peripheral events density by four times, and increased the central events density by eight times (Fig.3C). Inhibitions by XeC, RR, and XeC + RR of the ACh induced increases in peripheral events density (Fig.3C) were not different in AngII WT than in saline WT EC. Similarly, XeC and XeC + RR inhibitions of ACh induced increases in central events density (Fig.3C) were not different in AngII WT than in saline WT EC. However, the inhibition by RR of ACh induced increases in central events density (Fig.3C) was decreased in AngII WT relative to saline WT EC.

**Figure 4:**
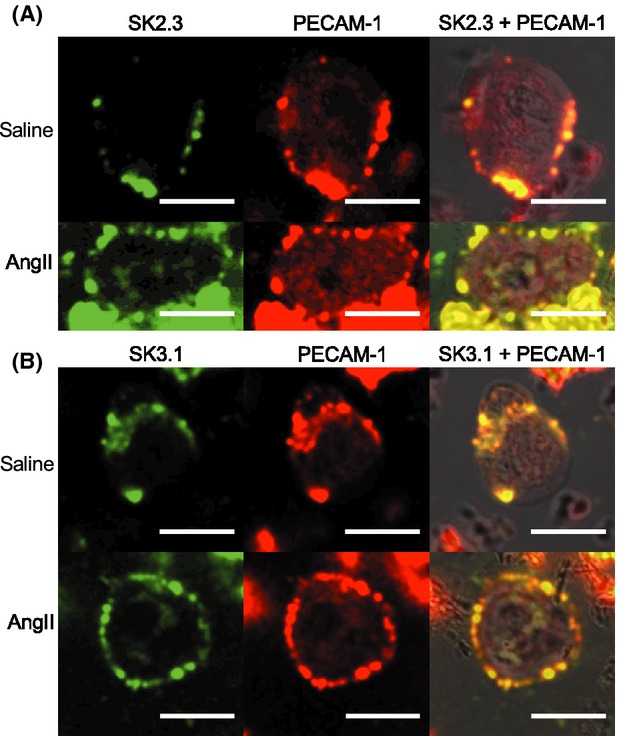
Expression of K_C__a_2.3, and K_C__a_3.1 expression in EC of small caliber arteries in healthy and diseased states. (A) Immunofluorescence detection of K_C__a_2.3 alone (green), PECAM-1 alone (red), and K_C__a_2.3 + PECAM-1 merged with BF images (yellow, K_C__a_2.3 + PECAM-1 overlap) in EC from PAR2 WT mice, after 14 days infusion with vehicle (saline) or AngII. (B) Immunofluorescence detection of K_C__a_3.1 alone (green), PECAM-1 alone (red), and K_C__a_3.1 + PECAM-1 merged with BF images (yellow, K_C__a_3.1 + PECAM-1 overlap) in WT saline and AngII EC. Fixed permeabilized EC from small caliber mesenteric arteries incubated with K_C__a_2.3- or K_C__a_3.1-, and PECAM-1 primary antibodies, and FITC- and Texas Red-conjugated secondary antibodies. White bar=10 *μ*m.

To characterize the expression of downstream Ca^2+^-sensitive targets of PAR2 mechanism in the isolated EC of small caliber arteries in the healthy and diseased states, we quantified eNOS-, K_Ca_2.3-, and K_Ca_3.1-antibodies immunofluorescence across the mid-plane of EC. eNOS was expressed throughout the cytoplasm, and colocalized with PECAM-1 at the periphery in saline WT and AngII WT EC (Fig.3D). Peripheral distribution of eNOS in AngII was reduced relative to saline WT EC (Fig.3D). K_Ca_2.3, and K_Ca_3.1 were expressed at peripheral sites colocalized with PECAM-1 in saline WT and AngII WT EC. K_Ca_2.3 (Fig.4A) and K_Ca_3.1 (Fig.4B) distributions were less uniform along the circumferences of EC, instead displaying punctate expression. K_Ca_2.3 and K_Ca_3.1 distributions in saline WT did not differ in AngII WT EC.

## Discussion

We investigated the mechanisms of Ca^2+^-release elicited by PAR2 activation in single EC from small caliber arteries in the healthy and diseased states. In EC, at baseline and during exposure to agonists, two types of Ca^2+^-release are identifiable; peripheral and central events, which are characterized by distinct spatial and temporal kinetic properties. PAR2-activating peptide concentration-dependently increased the densities and firing rates of peripheral and central events, indicating a direct control of intracellular Ca^2+^-release by the PAR2 activation pathway. The alteration of this Ca^2+^-release by XeC revealed that the IP_3_R is critical to the PAR2 Ca^2+^ signaling mechanisms. PAR2 activation of phospholipase C, which converts PIP_2_ to IP_3_, is well-established. However, our evidence that indicates there are two forms of events and both are mediated by IP_3_R, raises the question of how a single mediator produces two distinctly different Ca^2+^-release events.

Our Ca^2+^-imaging data indicate a specific distribution pattern of Ca^2+^-signal generators that produces the distinct peripheral versus central events. For instance, the cell volumes occupied by central events are much larger than peripheral events, suggesting that the molecular components of the Ca^2+^-handling function are different. We report kinetic properties for peripheral and central events that infer Ca^2+^-release (*t*_rise_) and Ca^2+^-uptake (*t*_1/2_) functions are different for each event type. IP_3_- and Ca^2+^-sensitivities differ between the three types of IP_3_R (Taylor et al. 2014) and a triple layer system of IP_3_R and ryanodine receptors models Ca^2+^-signaling in cardiac purkinje fibers (Haq et al. 2013). In small caliber arteries, IP_3_R distributed near their Ca^2+^-signals downstream target, that is, K_Ca_2.3 was widespread on the cell surface whereas K_Ca_3.1 localized to myoendothelial projections (Dora et al. 2008; Ledoux et al. 2008b). Therefore, these studies infer the potential for different pools of IP_3_R to associate with different Ca^2+^-sensitive targets.

A decrease in densities of peripheral and central events with TRPV channel inhibitor RR demonstrated that Ca^2+^ entry modifies intracellular IP_3_R-mediated Ca^2+^-release by PAR2. Ryanodine receptors are not expressed in native mouse mesenteric EC (Ledoux et al. 2008b) but could be inhibited by RR in cardiac cells. We would have expected an increase in Ca^2+^ within the cytosol rather than small decreases if the RR-mediated inhibition of mitochondrial calcium uniporter was contributing to Ca^2+^-dynamics in our EC preparations. Inhibiting Ca^2+^ entry via TRPV channels decreases Ca^2+^-stores, leading to lower capacity for Ca^2+^-release upon IP_3_R activation. During complete inhibition of IP_3_R and/or depleted intracellular stores, cooperative TRPV4 activation results in Ca^2+^-entry signals, called ‘sparklets’ (Sonkusare et al. 2012). Sparklets had amplitudes similar to the peripheral events in our study; however, the time-course for sparklets lasted several seconds. Our EC developed membrane blebs after 20 sec of laser illumination so we recorded data for shorter periods. In non-EC, PAR2 activation caused sensitization of TRPV Ca^2+^-entry (Grace et al. 2014). Although distribution of sparklets was widespread, K_Ca_3.1 activation was associated with the sparklets at myoendothelial projections (Sonkusare et al. 2014). In our study, central events had similar characteristics to Ca^2+^ events called ‘pulsars’ (Ledoux et al. 2008b), e.g., repeating frequency, fixed locations. However, it is uncertain that central events and pulsars are the same IP_3_R-mediated Ca^2+^-release, because EC isolation naturally results in losing the native EC abluminal–luminal polarity and architecture of an intact vessel, including the myoendothelial projections where pulsars were exclusively localized.

Although ACh elicited peripheral and central Ca^2+^-release events identical to those elicited by 2fly, agonist selective effects on total Ca^2+^-release function differentiated muscarinic receptors from PAR2 Ca^2+^-release mechanisms. We report that in EC, 2fly has greater total Ca^2+^-release activity than ACh. PAR2 activation elicited larger eNOS-independent vasodilation of small caliber arteries than ACh (McGuire et al. 2002). These observations raise the question of how two G*α*q-coupled receptors can produce different patterns of Ca^2+^ signals and appear to link tissue function to their selective Ca^2+^ signals. Heterogeneity in the mechanisms of endothelium-mediated vasodilation/hyperpolarization of arteries is well-described (McGuire et al. 2001). We propose that a cooperative relationship between PAR2 and IP_3_R may contribute to higher Ca^2+^-release activity, and in particular, proportionately higher peripheral Ca^2+^-release in proximity to K_Ca_2.3 and K_Ca_3.1 at the plasmalemma. In our study, PAR2 and IP_3_R were detected at the plasmalemma and more heterogeneously throughout the cytoplasm in EC. In EC of various rat arteries, expression of the three IP_3_R subtypes was described as heterogeneously distributed in EC and included distinct IP_3_R2 expression near the nucleus (Grayson et al. 2004). In permeabilized EC, PAR2 detection by B5-antibody was expected to colocalize with PECAM-1 at the plasmalemma and internal staining may reflect receptor reserves. GPCR expression in the nuclear envelope and nuclear Ca^2+^ signaling by GPCR have been described in vascular smooth, endothelial, and cardiac cells (Tadevosyan et al. 2012), so we have not ruled-out that PAR2 was activated at the nucleus.

By characterizing and comparing the Ca^2+^ dynamics in single EC from healthy and diseased state arteries, we found that PAR2 Ca^2+^-release mechanisms and PAR2 expression were unaffected in AngII EC. As previously reported, PAR2 vasodilation via the endothelium is preserved and involves primarily K_Ca_2.3 and K_Ca_3.1 in small caliber arteries from healthy animals and animals with endothelial dysfunction (McGuire et al. 2002; Chia et al. 2011). In our study, K_Ca_2.3 and K_Ca_3.1 expressions were not different between saline and AngII WT EC. These data indicate that the PAR2 vasodilation mechanism extending from Ca^2+^-release to K_Ca_2.3 and K_Ca_3.1 is preserved in the EC of diseased vasculature.

Since PAR2 Ca^2+^-release was preserved in AngII WT EC, validating dysfunction in single EC was an important part of our study. Previously with the AngII model, we reported results like others (Ryan et al. 2004) indicating a decreased ACh-mediated vasodilation of small mesenteric arteries associated with decreased eNOS activity (Chia et al. 2011). Here, ACh elicits Ca^2+^-signals in saline WT EC that decreased in AngII WT EC. While our data do not allow us to exclude other possible mechanisms, we interpret these data to suggest an upstream target closer to receptor activation is a site of lesion for muscarinic receptors in EC. We report that the distribution of eNOS was reduced in AngII WT EC, which is consistent with decreased eNOS-mediated vasodilation during endothelial dysfunction.

How our findings about reduced EC Ca^2+^ signals by ACh in the AngII model may be integrated with other proposed mechanisms is interesting for future study. We report that RR was ineffective as an inhibitor against ACh in EC after AngII infusion in vivo, but maintained inhibition against PAR2. Using the AngII model, EC, and arteries like those in our study, investigators reported decreased muscarinic receptor activation of TRPV4 and vasodilation, but unchanged maximum current densities for TRPV4 and K_Ca_3.1 in AngII EC (Sonkusare et al. 2014). Muscarinic receptor activation of TRPV4 was dependent on A-kinase anchoring protein-150 expression which decreased after AngII (Sonkusare et al. 2014). We speculate that A-kinase anchoring protein 150-dependency differentiates muscarinic receptors activated Ca^2+^ signaling from that of PAR2, and thus, may explain ACh susceptibility and PAR2 resistance to vasodilator dysfunction after chronic AngII.

In AngII WT EC, PAR2 Ca^2+^-release mechanisms were preserved as were the expressions of PAR2, IP_3_R, and several downstream targets of PAR2 Ca^2+^-signals, including, K_Ca_2.3 and K_Ca_3.1. PAR2 activation of K_Ca_2.3 and K_Ca_3.1 cause EC-dependent hyperpolarization that relaxes VSMC in small caliber arteries (McGuire et al. 2004a). Modulating the relaxation-contraction of such arteries alters peripheral resistance and cardiovascular function in vivo. PAR2-activating peptides administered acutely increased forearm blood flow in healthy human volunteers and administered in vivo decreased systolic blood pressures in unrestrained mice over a 14 day period (Robin et al. 2003; Hughes et al. 2013). PAR2 selective activation of peripheral IP_3_R-mediated Ca^2+^-signals and the sustained expression of K_Ca_2.3 and K_Ca_3.1, which are the primary mediators of PAR2 vasodilation, could rescue EC cardiovascular function, such as modulating VSMC contraction, blood flow, and blood pressure, in the diseased state.

### Limitations

PAR2 agonists cause global cytosolic Ca^2+^ transients in EC in cultured cell monolayers. Here we report the temporal and spatial kinetic characteristics of two types of distinct and specific Ca^2+^ release events that occurred in different locations within EC and at agonist concentrations relevant to vasodilation of intact arteries (Chia et al. 2011). We determined not only the kinetic properties of each event type, but also identified receptor-dependent pathways controlling the regional Ca^2+^-releases. We resolved Ca^2+^-signals at the single EC level, using freshly isolated preparations and 2D spinning disk CM techniques. EC dimensions are so small that in each confocal plane, one frame of 2D fluorescence data captured about 25% of the EC volume (4 *μ*m thickness). The 2D confocal video frame rate was sufficiently fast to capture Ca^2+^-events reported in EC. We show EC receptor-specific dysfunction at the level of whole arteries extends to the level of single EC. On one hand, the single-cell level provides a level of cellular specificity for investigation and facilitates translating experiments with human cells. On the other hand, the single-cell level restricted the scope to EC, and thus, future studies may consider PAR2 Ca^2+^ signaling in the context of EC to EC and EC to VSMC.

## Conclusions

PAR2 Ca^2+^-release in EC of small caliber arteries in the healthy state comprised two distinct specific Ca^2+^ events. In EC of small caliber arteries in a diseased state, PAR2 mechanisms of IP3R-mediated Ca^2+^-release were preserved. Further studies of PAR2-selective Ca^2+^-release for eliciting pathological and/or normal EC functions in tissues and in vivo are warranted.

## Abbreviations

2D: 2-dimension
ACh: acetylcholine
AKAP150: A-kinase anchoring protein 150
AngII: angiotensin-II
BF: bright field
CM: confocal microscopy
EC: endothelial cell
ECIB: EC isolation buffer
EGTA: ethylene glycol tetraacetic acid
eNOS: endothelial or nitric oxide synthase 3
FITC: fluorescein isothiocyanate
FWHM: full width at half maximum
HEPES: 4-(2-hydroxyethyl)-1-piperazineethanesulfonic acid hemisodium salt
IP_3_R: inositol 1,4,5-trisphosphate receptor
K_Ca_2.3: small-conductance Ca^2+^-activated potassium channel KCNN3
K_Ca_3.1: intermediate-conductance Ca^2+^-activated potassium channel KCNN4
KO: PAR2 transgenic knockout mice
PAR2: proteinase-activated receptor 2
PBS: phosphate buffered solution
PECAM-1: platelet endothelial cell adhesion molecule-1
RR: ruthenium red
t_1/2_: time-to-fall
*t*_rise_: time-to-rise
TRPV: transient receptor potential vanilloid channel
VSMC: vascular smooth muscle cell
WT: wild-type
XeC: xestospongin C
